# GDP-D-mannose epimerase regulates male gametophyte development, plant growth and leaf senescence in *Arabidopsis*

**DOI:** 10.1038/s41598-017-10765-5

**Published:** 2017-09-04

**Authors:** Tiancong Qi, Zhipeng Liu, Meng Fan, Yan Chen, Haixia Tian, Dewei Wu, Hua Gao, Chunmei Ren, Susheng Song, Daoxin Xie

**Affiliations:** 1 Tsinghua-Peking Center for Life Sciences, MOE Key Laboratory of Bioinformatics, School of Life Sciences, Tsinghua University, Beijing, 100084 China; 2 State Key Laboratory of Grassland Agro-ecosystems, College of Pastoral Agriculture Science and Technology, Lanzhou University, Lanzhou, 730020 China; 3 College of Bioscience and Biotechnology, Crop Gene Engineering Key Laboratory of Hunan Province, Hunan Agricultural University, Changsha, Hunan 410128 China; 4 Beijing Key Laboratory of Plant Gene Resources and Biotechnology for Carbon Reduction and Environmental Improvement, College of Life Sciences, Capital Normal University, Beijing, 100048 China

## Abstract

Plant GDP-D-mannose epimerase (GME) converts GDP-D-mannose to GDP-L-galactose, a precursor of both L-ascorbate (vitamin C) and cell wall polysaccharides. However, the genetic functions of *GME* in *Arabidopsis* are unclear. In this study, we found that mutations in *Arabidopsis GME* affect pollen germination, pollen tube elongation, and transmission and development of the male gametophyte through analysis of the heterozygous *GME*/*gme* plants and the homozygous *gme* plants. *Arabidopsis gme* mutants also exhibit severe growth defects and early leaf senescence. Surprisingly, the defects in male gametophyte in the *gme* plants are not restored by L-ascorbate, boric acid or GDP-L-galactose, though boric acid rescues the growth defects of the mutants, indicating that GME may regulate male gametophyte development independent of L-ascorbate and GDP-L-galactose. These results reveal key roles for *Arabidopsis GME* in reproductive development, vegetative growth and leaf senescence, and suggest that *GME* regulates plant growth and controls male gametophyte development in different manners.

## Introduction

L-Ascorbate (vitamin C), a common natural water-soluble antioxidant in plants^1^, affects plant growth^2, 3^, leaf senescence^4^ and photosynthesis^5^, and it regulates plant responses to pathogen infection^6^ and various abiotic stresses^7–10^. L-Ascorbate is mainly biosynthesised from D-glucose via sequential enzymatic reactions in the L-galactose (L-Gal) pathway^11, 12^. GDP-D-mannose epimerase (GME) catalyses the conversion of GDP-D-mannose to GDP-L-Gal and GDP-L-gulose, which is a key step in the L-ascorbate pathway^13–15^. In addition, GDP-L-Gal acts as a precursor of cell wall polysaccharides such as rhamnogalacturonan II (RGII), which is a crucial polysaccharide component of pectin^16, 17^.

*GME* is the most conserved gene in the ascorbate biosynthesis pathway^18^. Tomato contains two homologous GMEs^19^, while most plants such as *Arabidopsis* ^13^, rice^20^, *Medicago* ^21^ and peach^22^ have only one copy of *GME* ^20^. In this study, we isolated *Arabidopsis* T-DNA insertion mutants of *GME* to examine its biological functions. We found that *Arabidopsis GME* is vital for plant vegetative growth, leaf senescence and male gametophyte development and transmission.

## Results

### The expression pattern and subcellular localisation of *Arabidopsis GME*

**Figure 1:**
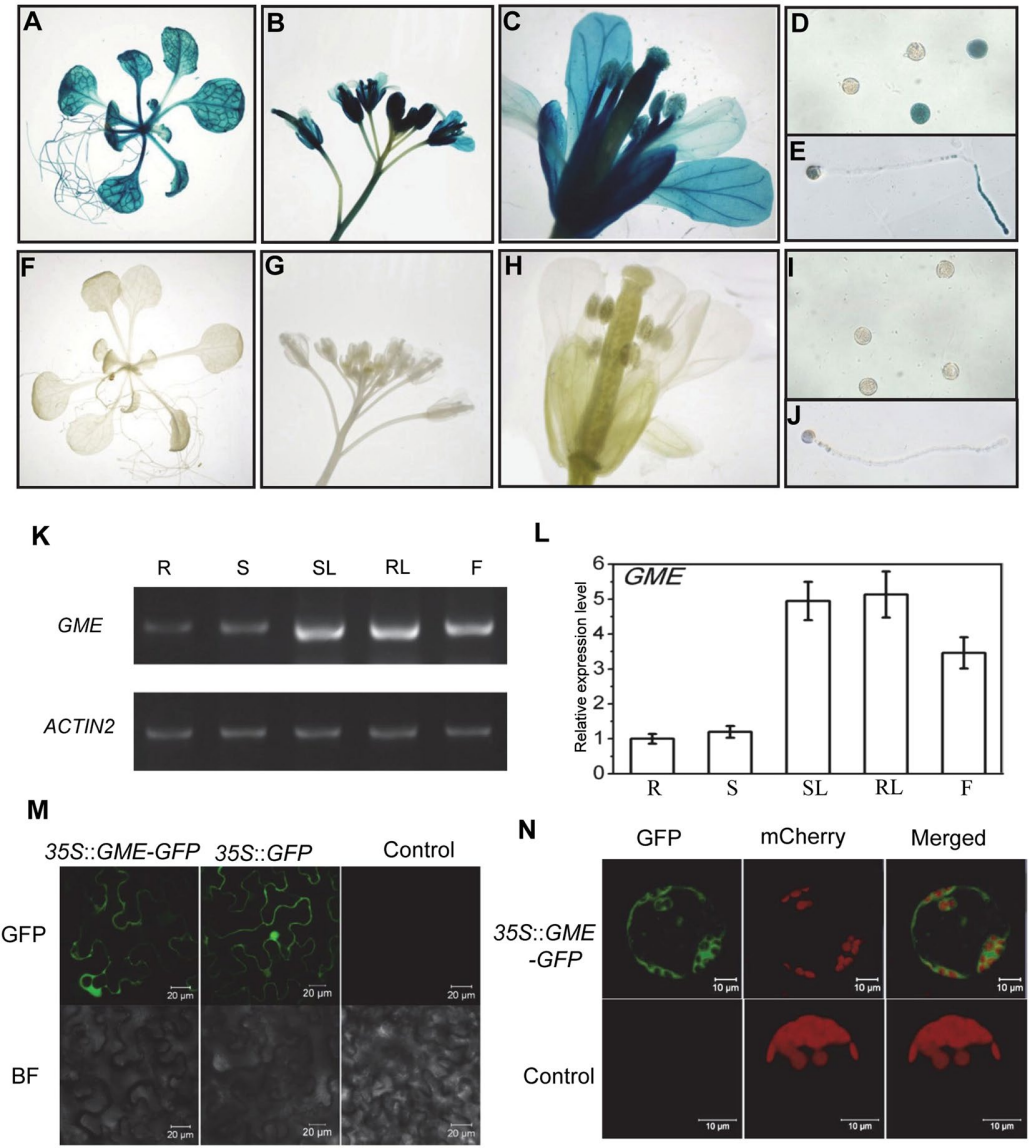
Expression pattern and subcellular localisation of *GME* in *Arabidopsis*. (**A**–**J**) Histochemical GUS activity in seedlings (**A**), inflorescences (**B**), flowers (**C**), pollen grains (**D**) and pollen tubes (**E**) from T1 transgenic *Arabidopsis* expressing the *GUS* reporter gene under the control of the GME promoter (*P*_*GME*_::*GUS*) with GUS activity in wild type (**I**,**J**) as a control. (**K**) and (**L**) RT-PCR analysis (**K**) and real-time PCR analysis (**L**) of the *GME* expression level in *Arabidopsis* roots (**R**), stems (**S**), stem leaves (SL), rosette leaves (RL) and flowers (**F**). *ACTIN2* was used as the normalisation or internal control. (**M**) Subcellular localisation of GME in epidermal cells from *N*. *benthamiana* leaves. GME*-*fused GFP driven by the 35S promoter (*35S::GME-GFP*) and *35S::GFP* were expressed in *N*. *benthamiana* leaves, respectively. GFP fluorescence was detected 50 h after infiltration. Infiltration with buffer was used as a negative control. BF, bright field. (**N**) *Arabidopsis* protoplasts transformed without (Control) or with *35S::GME-GFP*. Red fluorescence indicates chloroplasts.

We generated transgenic plants harbouring the GUS reporter driven by the *GME* promoter (*P*_*GME*_::*GUS*) to analyse the *GME* expression pattern in *Arabidopsis*. GUS activity was detected in roots, leaves, stems and inflorescences, implying that *GME* plays key roles in growth and development (Fig. 1A,B,F and G). Moreover, the floral organs of the plants (sepals, petals, stamens and carpels), including pollen grains and pollen tubes, exhibited GUS activity, suggesting that *GME* affects reproductive development (Fig. 1C–E and H–J). We also examined the *GME* expression pattern by reverse transcription PCR (RT-PCR) and quantitative real-time PCR (qRT-PCR), and found that the *GME* expression level was high in rosette leaves, stem leaves and flowers, and low in roots and stems (Fig. 1K and L). These results indicate that *GME* may affect various aspects of plant growth and development.

We next transiently expressed GME fused with GFP (GME-GFP) in *Nicotiana benthamiana* leaves to observe the subcellular localisation of GME. Strong GFP fluorescence was detected in the cytoplasm of epidermal cells (Fig. 1M); fluorescence due to GFP alone (as a control) was detected in both the cytoplasm and nucleus (Fig. 1M). To confirm the subcellular localisation of GME, we transiently expressed GME-GFP in *Arabidopsis* protoplasts and observed GFP fluorescence only in the cytoplasm (Fig. 1N). These results demonstrate that GME is localised in the cytoplasm (Fig. 1M and N).

### *Arabidopsis GME* controls male gametophyte transmission

**Figure 2:**
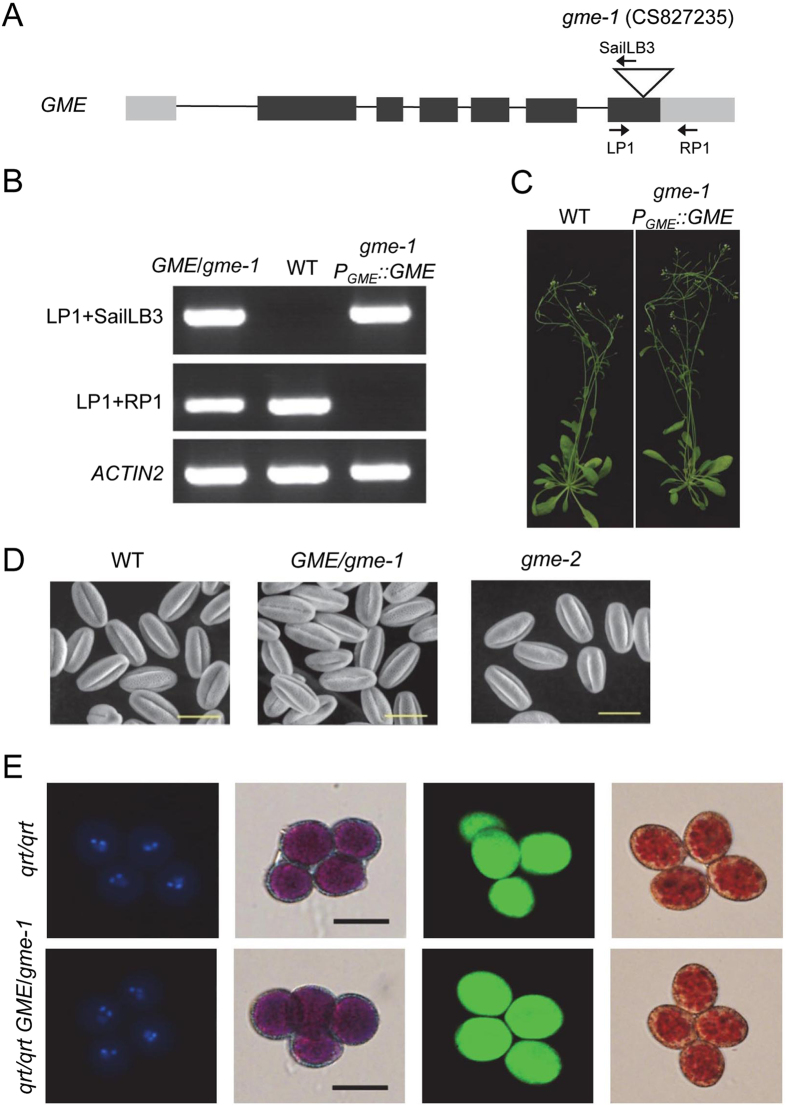
Characterisation and genetic complementation of the *gme-1* mutation and an analysis of tricellular pollen grains. (**A**) Schematic diagram showing the T-DNA insertion in *GME* and the core structure of the *P*_*GME*_::*GME* vector (*GME* under the control of the *GME* promoter). The grey rectangle, black rectangle and triangle represent the UTR, exon and T-DNA insertion site, respectively. The primers indicated by arrows were used to identify the genetic background of the *gme-1* mutants in (**B**). (**B**) Genotyping of *GME*/*gme-1*, Col-0 wild type (WT), and transgenic *P*_*GME*_::*GME* in a *gme-1* background. *gme-1 P*_*GME*_::*GME* was generated by transforming *P*_*GME*_::*GME* into *GME*/*gme-1* heterozygous plants. The primer pairs LP1/SailLB3 and LP1/RP1, respectively, are specific for the *gme-1* T-DNA insertion and WT GME. *ACTIN2* PCR products were used as a control. (**C**) Six-week-old Col-0 WT and *gme-1 P*_*GME*_::*GME* plants. (**D**) Environmental scanning electron microscopy of pollen grains at floral stage 13 from WT, *GME*/*gme-1* and *gme-2* plants. Bars = 20 µm. (**E**) The *gme-1* mutation does not affect male meiosis, mitosis, pollen viability or pollen vacuole development at the tricellular stage. The panels from left to right show tetrad pollen grains at the tricellular stage stained, respectively, with DAPI, Alexander’s stain, fluorescein diacetate/propidium iodide and neutral red. Bars = 20 µm.

We next examined two T-DNA insertion mutants of *GME*: *gme-1* (CS827235, with a T-DNA insertion in the last exon of *GME*) and *gme-2* (Salk_008960, with a T-DNA insertion in the 5′-untranslated region [UTR] of *GME*) (Fig. 2A and Supplemental Fig. 1). Interestingly, we obtained *gme-2* homozygotes, but not *gme-1* homozygotes. The progeny of the *GME*/*gme-1* heterozygotes included only wild-type (WT) plants and *GME*/*gme-1* heterozygotes, and the siliques of *GME*/*gme-1* appeared normal without any aborted seeds (Supplemental Fig. 2), implying that the T-DNA insertion in *gme-1* attenuated transmission of the male gametophyte, but not embryonic development.

We next performed reciprocal crosses to determine which type of gametophyte development was affected in *GME*/*gme-1*. When the stigmas of *GME*/*gme-1* heterozygotes were pollinated with WT pollen grains, the progeny segregation ratio (*GME*/*GME*:*GME*/*gme-1*) was identical to the expected 1:1 (Table 1). Conversely, when pollen grains from *GME*/*gme-1* heterozygotes were crossed onto WT stigmas, all progeny were wild type (Table 1), suggesting that *gme-1* is a null mutation that leads to complete failure of transmission of the male gametophyte, but not the female gametophyte. We also transformed *GME*/*gme-1* heterozygotes with the coding sequence of *GME* driven by its native promoter (*P*_*GME*_::*GME*) to obtain *P*_*GME*_::*GME* transgenic plants in a *gme-1* background (*gme-1 P*_*GME*_::*GME*) (Fig. 2B and C), demonstrating that *GME* complemented the transmission defect caused by the *gme-1* mutation.

**Table 1:** Genetic transmission analysis of *gme* alleles.

Parental Genotype	T-DNA^+^	T-DNA^−^	TE (%)
*GME*/*gme-1* self-fertilized	111	116	48.9
*GME*/*gme-2* self-fertilized	101	81	55.5
♀ *GME*/*gme-1* × *♂*WT	168	161	51.0
♀ WT × *♂ GME*/*gme-1*	0	150	0
♀ *GME*/*gme-2* × *♂*WT	94	100	48.5
♀ WT × *♂ GME*/*gme-2*	35	291	10.7

Progeny containing T-DNA insertion were identified by PCR. Transmission efficiency (TE) = Number of progeny with T-DNA insertion/Number of progeny ×100. WT, Col-0 wild type.

In reciprocal crosses performed with *GME*/*gme-2*, when the stigmas of *GME*/*gme-2* heterozygotes were pollinated with WT pollen grains, the progeny exhibited a 1:1 segregation ratio (*GME*/*GME*:*GME*/*gme-2*) (Table 1). However, when pollen grains from *GME*/*gme-2* plants were applied to WT stigmas, the progeny segregation ratio (*GME*/*GME*:*GME*/*gme-2*) was about 1:0.12 (Table 1), suggesting that *gme-2* is a strong mutation that dramatically reduces the transmission efficiency of the male gametophyte, but that it’s not a null mutation. Consistent with this conclusion, the progeny segregation ratio (*gme-2*/*gme-2* and *GME*/*gme-2*:*GME*/*GME*) from *GME*/*gme-2* plants was about 1.25:1, which is a clear reduction compared with a normal 3:1 segregation ratio (Table 1). We obtained only 15 *gme-2* homozygotes from 182 progeny of a *GME*/*gme-2* heterozygote (8.2%), which is much less than expected (25%).

Taken together (Table 1, Fig. 2A–C and Supplemental Figs 1 and 2), these results show that *Arabidopsis GME* is essential for male gametophyte transmission.

### *GME* is required for pollen germination and pollen tube elongation

In *Arabidopsis* anthers, the microspore mother cells undergo sequential meiosis and mitosis to form tricellular pollen grains containing two sperm cell nuclei and one vegetative cell nucleus. Pollen grains from dehisced anthers are released onto the stigma of a carpel and germinate to form pollen tubes, which elongate and go through the stigma and transmitting tract, thereby delivering the two sperm cells to an ovary for double fertilisation^23–26^. Any disruption in this process will lead to failed male gametophyte transmission.

We next explored in which stages *GME* regulates male gametophyte development. Observation of the surface of mature pollen grains from WT, *GME*/*gme-1* heterozygous and *gme-2* homozygous plants by environmental scanning electron microscopy showed that all of the pollen grains from the *gme* mutants were oval-shaped with long indented lines on their surface as in wild type (Fig. 2D).

We next crossed *GME*/*gme-1* plants with the *quartet1* (*qrt*) mutant, which releases unseparated pollen tetrads derived from a single pollen mother cell^27^, to generate *qrt*/*qrt GME*/*gme-1* plants. 4′,6-Diamidino-2-phenylindole (DAPI) staining showed that all four pollen grains of the *qrt*/*qrt GME*/*gme-1* tetrads contained three nuclei (Fig. 2E). Alexander staining, fluorescein diacetate/propidium iodide double staining, and neutral red staining showed that all of the pollen grains from *qrt*/*qrt GME*/*gme-1* were viable with normal pollen vacuoles (Fig. 2E). These results (Fig. 2E) demonstrate that *GME* does not affect the formation of tricellular pollen grains.

**Figure 3:**
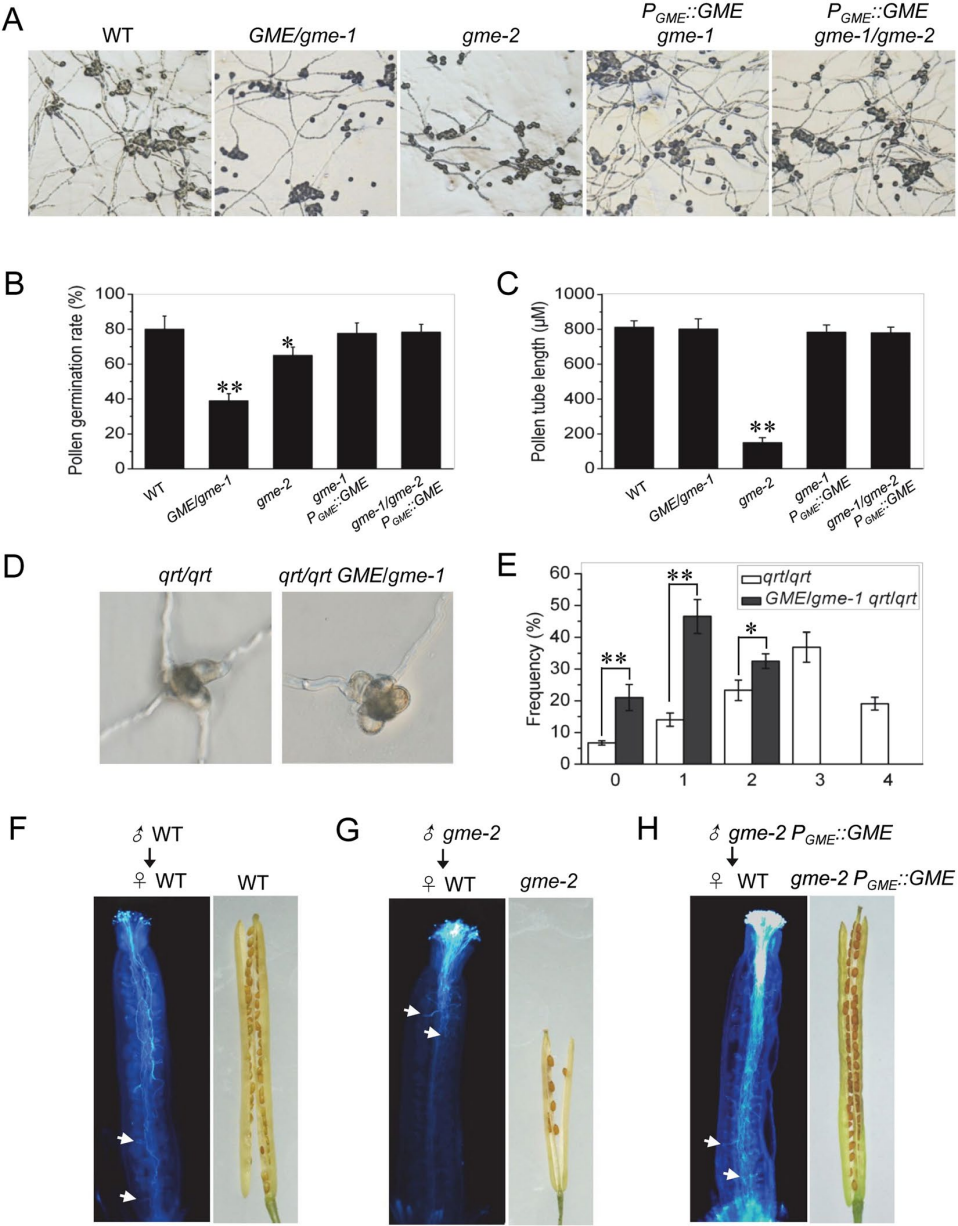
*GME* is required for pollen germination and pollen tube elongation. (**A**–**C**) *In vitro* pollen germination (**A**), statistical analysis of the pollen germination rate (**B**) and pollen tube length (**C**) in Col-0 WT, *GME*/*gme-1*, *gme-2*, *gme-2 P*_*GME*_::*GME* and *gme-1*/*gme-2 P*_*GME*_::*GME* plants. Error bars represent the standard error (SE; n = 3). Asterisks represent Student’s *t-*test significance compared with wild type (*P < 0.05, **P < 0.01). (**D**) *In vitro* pollen germination in *qrt*/*qrt* and *qrt*/*qrt GME*/*gme-1*. (**E**) Frequency (%) of *qrt*/*qrt* and *qrt*/*qrt GME*/*gme-1* tetrads with the indicated numbers (0, 1, 2, 3 or 4) of germinated pollen grains. Error bars represent the SE (n = 3). Asterisks represent Student’s *t*-test significance between pairs indicated with brackets (*P < 0.05, **P < 0.01). (**F**–**H**) Aniline blue staining of pollen tubes showing that WT (**F**) and *gme-2 P*_*GME*_::*GME* (H) pollen tubes germinated for 16 h in WT female organs could reach the base of the transmitting tract, while *gme-2* pollen tubes (**G**) could only reach half of the transmitting tract. Consistently, mature WT (**F**) and *gme-2 P*_*GME*_::*GME* (**H**) siliques were full of seeds, while *gme-2* siliques (**G**) contained few seeds in the upper part of the silique. White arrows indicate pollen tubes.

*In vitro* pollen germination assays were performed to explore whether the processes that occur after tricellular pollen grain formation are affected in *gme* mutants. As shown in Fig. 3A and B, the pollen grains from *gme-1* heterozygous and *gme-2* homozygous plants exhibited reduced germination rates (~39% for *GME*/*gme-1* and ~65% for *gme-2*) compared with wild type (~80%). Consistently, a maximum of four pollen grains from the *qrt*/*qrt* pollen tetrads could germinate, while no more than two pollen grains from the *qrt*/*qrt GME*/*gme-1* pollen tetrads could germinate (Fig. 3D and E). Thus, *GME* is required for pollen germination.

Moreover, the pollen tube length in *gme-2* homozygous plants was much shorter than that in wild type (Fig. 3A and C), indicating that *GME* is required for pollen tube elongation. To confirm this conclusion, we performed *in vivo* pollen tube growth experiments. WT pollen tubes could reach the bottom of the transmitting tract (Fig. 3F), and WT siliques were consistently filled with seeds (Fig. 3F). In contrast, the pollen tubes of *gme-2* plants could reach no more than half the length of the transmitting tract, and mature *gme-2* siliques possessed few seeds that were mainly located in the upper part of the siliques (Fig. 3G).

Taken together (Fig. 3), these results demonstrate that *GME* is required for pollen germination and pollen tube elongation. In support of this conclusion, genetic complementation experiments showed that *GME* with its native promoter (*P*_*GME*_::*GME*) could rescue *in vitro* pollen germination, pollen tube elongation, *in vivo* pollen tube elongation and seed setting in *gme-2* plants and in *gme-1*/*gme-2*, which was generated by crossing *GME*/*gme-1* with *gme-2* (Fig. 3A–C and H).

### Defective male gametophyte development in gme mutant plants is not due to an ascorbate deficiency

**Figure 4:**
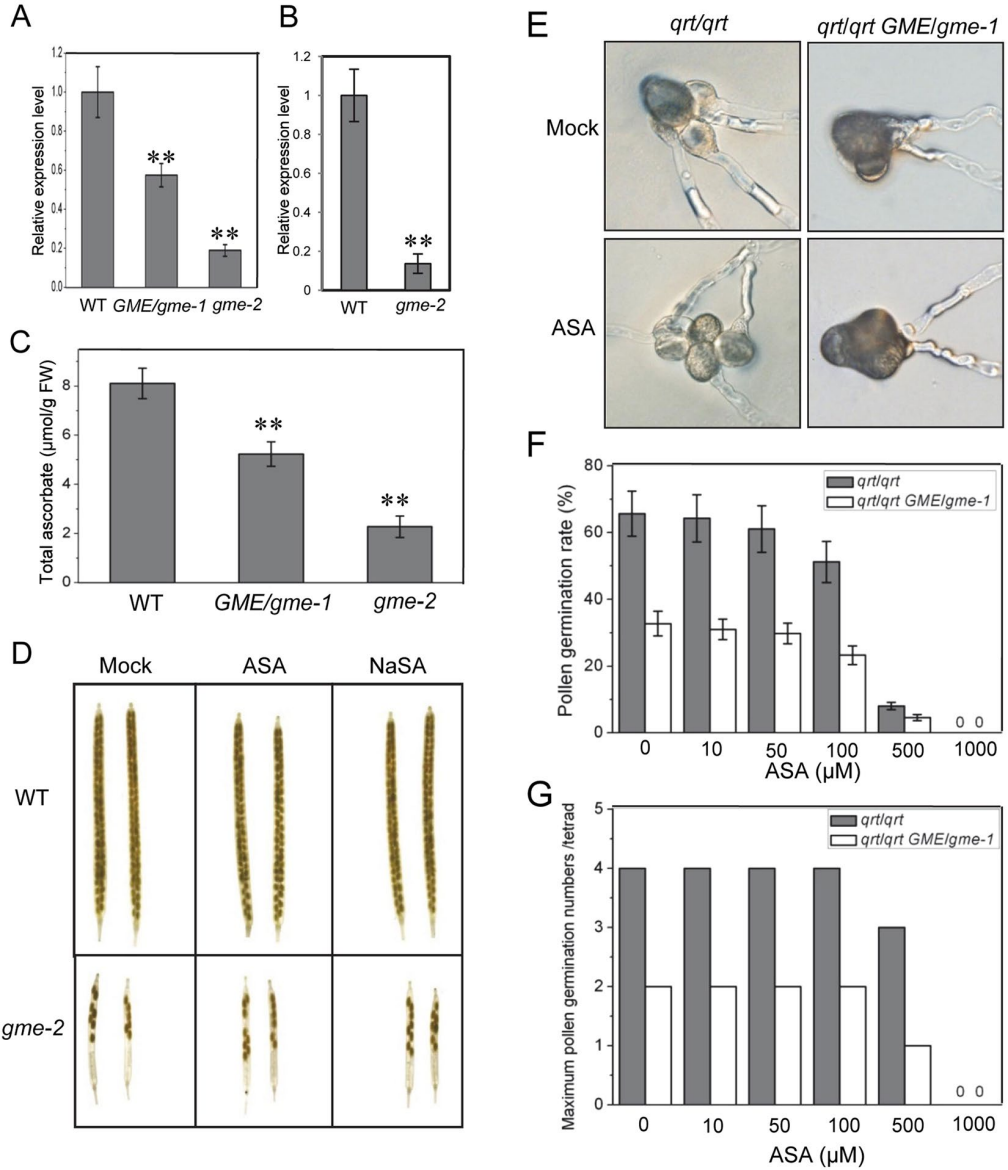
ASA or NaSA application cannot rescue the fertility of *gme-2*. (**A**) Real-time PCR analysis of the *GME* expression levels in WT, *GME*/*gme-1* heterozygous and *gme-2* homozygous plants. *ACTIN2* was used as the internal control. Error bars represent the SE (n = 3). Asterisks represent Student’s *t*-test significance compared with wild type (**P < 0.01). (**B**) Real-time PCR analysis of *GME* in pollen grains at floral stage 13 from WT and *gme-2* homozygous plants. (**C**) Total ascorbate contents in leaves from 5-week-old WT, *GME*/*gme-1* and *gme-2* plants. FW, fresh weight. Error bars represent the SE (n = 3). (**D**) Inflorescences from 6-week-old WT and *gme-2* plants were treated with mock, 1 mM ASA or 1 mM NaSA for 10 days, and the derived siliques were imaged. (**E**) *In vitro* pollen germination of *qrt*/*qrt* and *qrt*/*qrt GME*/*gme-1* treated with mock or 50 µM ASA. (**F**) Pollen germination rates in *qrt*/*qrt* and *qrt*/*qrt GME*/*gme-1* treated with 0, 10, 50, 100, 500 or 1000 µM ASA, respectively. Error bars represent the SE (n = 3). (**G**) The maximum number of germinated pollen grains per tetrad of *qrt*/*qrt* and *qrt*/*qrt GME*/*gme-1* in (**E**).

As GME is a key enzyme in L-ascorbate biosynthesis, we next explored whether the defects in male gametophyte development in the *gme* mutant plants was due to a deficiency in L-ascorbate. qRT-PCR analysis confirmed that *GME* expression was reduced in *GME*/*gme-1* and *gme-2* plants (Fig. 4A) and decreased in *gme-2* pollen grains (Fig. 4B). The ascorbate contents in *GME*/*gme-1* and *gme-2* plants were reduced to 64% and 28% of the WT level, respectively (Fig. 4B), demonstrating that ascorbate biosynthesis was decreased in these *Arabidopsis gme* mutants.

We next investigated whether the application of ascorbate could restore male gametophyte development in *gme* plants. The inflorescences of WT and *gme-2* plants were treated with L-ascorbic acid (ASA) and sodium ascorbate (NaSA), respectively. WT plants exhibited good fertility when treated without or with ASA or NaSA, while male fertility in *gme-2* could not be rescued by ASA or NaSA (Fig. 4D). Thus, ascorbate application could not rescue male gametophyte development in *gme-2*.

*In vitro* pollen germination assays were performed to detect whether ascorbate could rescue pollen germination in *qrt*/*qrt GME*/*gme-1*. As shown in Fig. 4E–G, ASA application could not recover the pollen germination rate in *GME*/*gme-1*, and it even inhibited pollen germination at high concentrations. Similar results were obtained for NaSA (data not shown). These results indicate that the defects in male gametophyte development in *gme* plants cannot be attributed to an ascorbate deficiency.

We next analysed mutants of the ascorbate biosynthetic genes *VTC2* and *VTC5*, which encode two GDP-L-Gal phosphorylases that function redundantly to control *Arabidopsis* ascorbate biosynthesis^2^, in order to verify that an ascorbate deficiency does not affect male gametophyte development. The double mutant *vtc2 vtc5* contained only ~22% of the WT level of ascorbate, and it exhibited severe growth defects (Supplemental Fig. 3), demonstrating that ascorbate biosynthesis in *vtc2 vtc5* was severely blocked. We also used *vtc2*/*vtc2 VTC5*/*vtc5* and *vtc5*/*vtc5 VTC2*/*vtc2* plants that were homozygous for one allele and heterozygous for the other to perform reciprocal crosses with wild type. As shown in Supplemental Table 1, regardless of whether WT plants or mutants were used as recipients, gametophyte transmission was unaffected, suggesting that the abolishment of ascorbate biosynthesis by the mutation of both *vtc2* and *vtc5* does not affect male gametophyte development.

Taken together (Fig. 4 and Supplemental Table 1), our data demonstrate that an ascorbate deficiency is not responsible for the defects in male gametophyte development observed in *gme* mutant plants.

### Boric acid and GDP-L-Gal cannot restore pollen germination and pollen tube growth in *gme* mutant plants

A previous study showed that the growth defects in *GME*-silenced tomato plants could be rescued by the application of boric acid, which promotes the boron-mediated *in muro* cross-linking of cell wall polysaccharides, but not by ascorbate^28^. We thus explored whether boric acid could rescue male gametophyte development in our *gme* mutant plants. *In vitro* pollen germination assays using *qrt*/*qrt* and *qrt*/*qrt GME*/*gme-1* supplied with different concentrations of boric acid showed that *qrt*/*qrt* displayed high pollen germination rates (~65–67%), while *qrt*/*qrt GME*/*gme-1* treated with different concentrations of boric acid exhibited low germination rates (~31–33%) (Supplemental Fig. 4A and B). These findings suggest that boric acid cannot restore pollen germination in *qrt*/*qrt GME*/*gme-1*.

Next, inflorescences from WT and *gme-2* plants were treated with boric acid. Regardless of whether they were treated with or without boric acid, the WT siliques were large and full of seeds while *gme-2* produced small siliques with few seeds (Supplemental Fig. 4C), indicating that boric acid supplementation could not restore male gametophyte development and fertility in *gme-2*.

As GDP-L-Gal is a precursor of cell wall polysaccharides (e.g., RGII)^19^, we also tested whether the application of GDP-L-Gal could restore pollen germination in *GME*/*gme-1*. Our results indicate that GDP-L-Gal was unable to recover pollen germination in *qrt*/*qrt GME*/*gme-1* (Supplemental Fig. 4D).

Taken together, these data (Supplemental Fig. 4) demonstrate that treatment with boric acid or GDP-L-Gal cannot restore male gametophyte development in *gme* mutants.

### Growth defects in *gme* mutants

**Figure 5:**
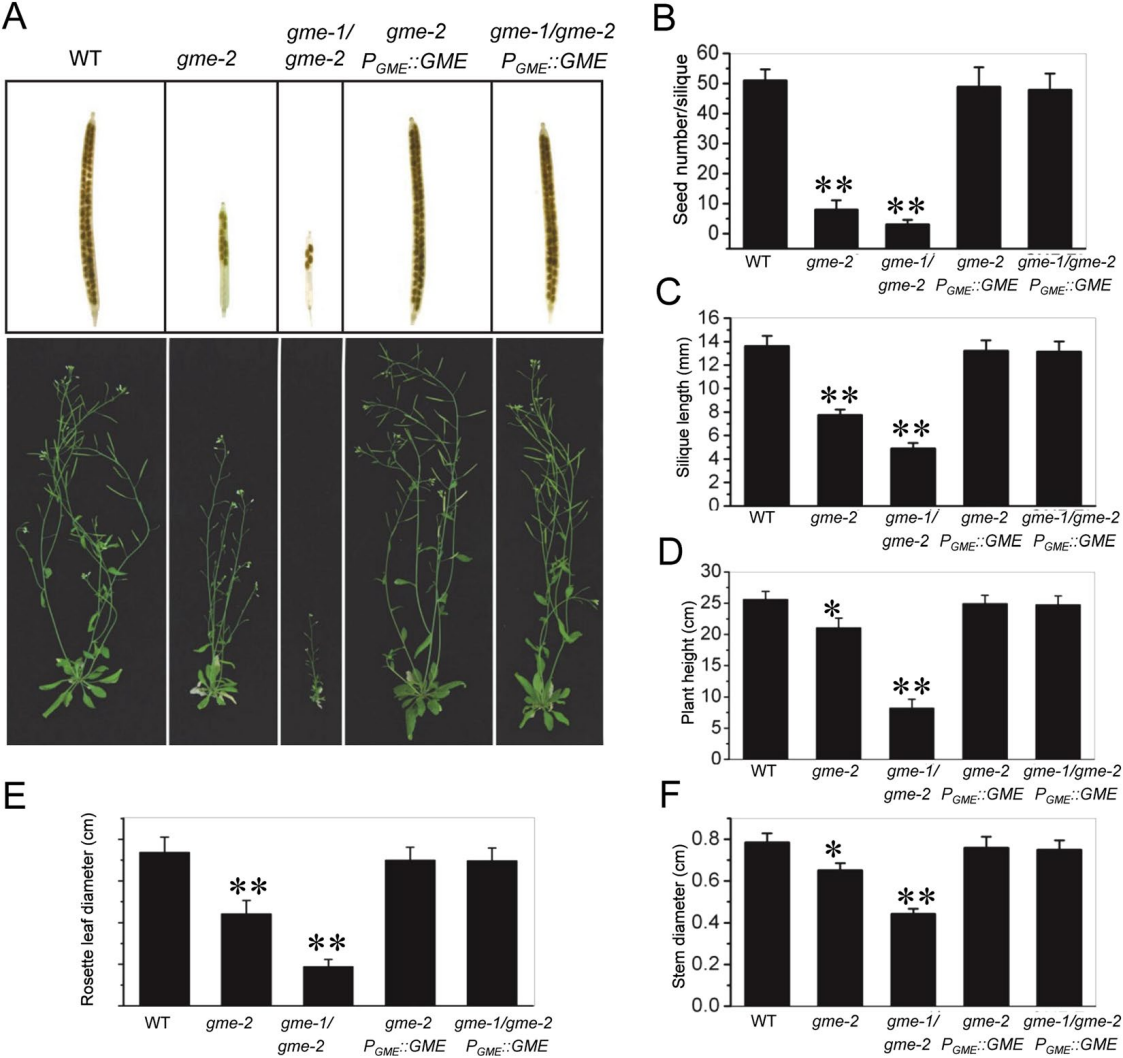
Growth defects and genetic complementation of the *gme-2* and *gme-1*/*gme-2* mutants. (**A**) Morphology of 6-week-old WT, *gme-2*, *gme-1*/*gme-2*, gme-2 *P*_*GME*_::*GME* and *gme-1*/*gme-2 P*_*GME*_::*GME* plants. (**B**–**F**) Histograms showing the seed number per silique (**B**), silique length (**C**), height (**D**), rosette leaf diameter (**E**) and stem diameter (**F**) of the plants in (**A**). Asterisks represent Student’s *t*-test significance compared with wild type (*P < 0.05, **P < 0.01).

We next investigated whether *Arabidopsis GME* regulates growth. As shown in Fig. 5, *gme-2* homozygous plants exhibited retarded growth in terms of their rosette leaves, height, stem diameter and fertility (e.g., silique length and seed number per silique; Fig. 5). Compared with *gme-2*, *gme-1*/*gme-2* plants showed much more severe growth defects, including a dramatically reduced rosette leaf size, thinner stems, shorter siliques and fewer seeds (Fig. 5 and Supplemental Fig. 1). The growth defects of *gme-2* and *gme-1*/*gme-2* could be restored by genetic complementation with *GME* (Fig. 5). Thus, *GME* plays important roles in vegetative growth.

### The growth defects in *gme* can be rescued by boric acid but not ascorbate

**Figure 6:**
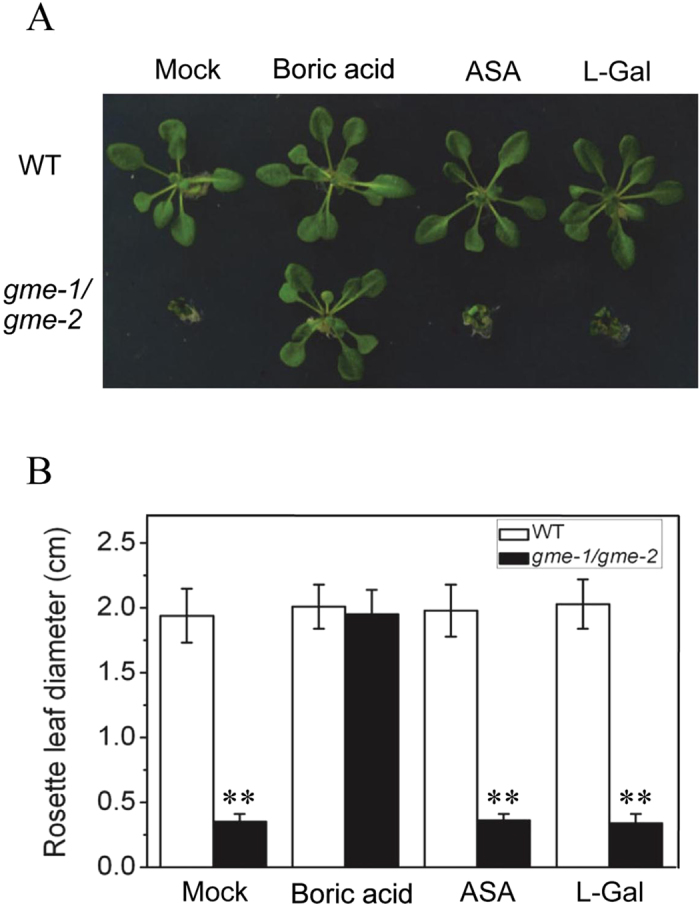
The growth defects of *gme-1*/*gme-2* can be rescued by boric acid. (**A**) Three-week-old WT and *gme-1*/*gme-2* seedlings grown on boric acid-free MS medium supplied without (Mock) or with 100 µM boric acid, 100 µM ASA or 100 µM L-Gal. (**B**) Rosette leaf diameter of the seedlings shown in (**A**). Error bars represent the SE (n = 8). Asterisks represent Student’s *t-*test significance compared with wild type (**P < 0.01).

To explore the reason for the growth defects of the *Arabidopsis gme* mutants, we treated *gme-1*/*gme-2* plants with boric acid, ASA or L-Gal, respectively. As shown in Fig. 6A and B, the growth defects of the *gme-1*/*gme-2* mutant could be rescued by boric acid, but not by ASA or L-Gal, suggesting that the growth defects of the *gme-1*/*gme-2* mutant were due to reduced *in muro* cross-linking of cell wall polysaccharides. On the other hand, the severe growth defects of the ascorbate-deficient mutant *vtc2 vtc5* could be recovered by ASA and L-Gal, but not by boric acid (Supplemental Fig. 5), suggesting that the growth defects of the *vtc2 vtc5* double mutant were due to an ascorbate deficiency rather than *in muro* cross-linking of cell wall polysaccharides.

In conclusion, these results (Fig. 6 and Supplemental Fig. 5) demonstrate that the growth defects of *gme* mutants are caused by reduced *in muro* cross-linking of cell wall polysaccharides.

### Early leaf senescence in *gme* mutants

**Figure 7:**
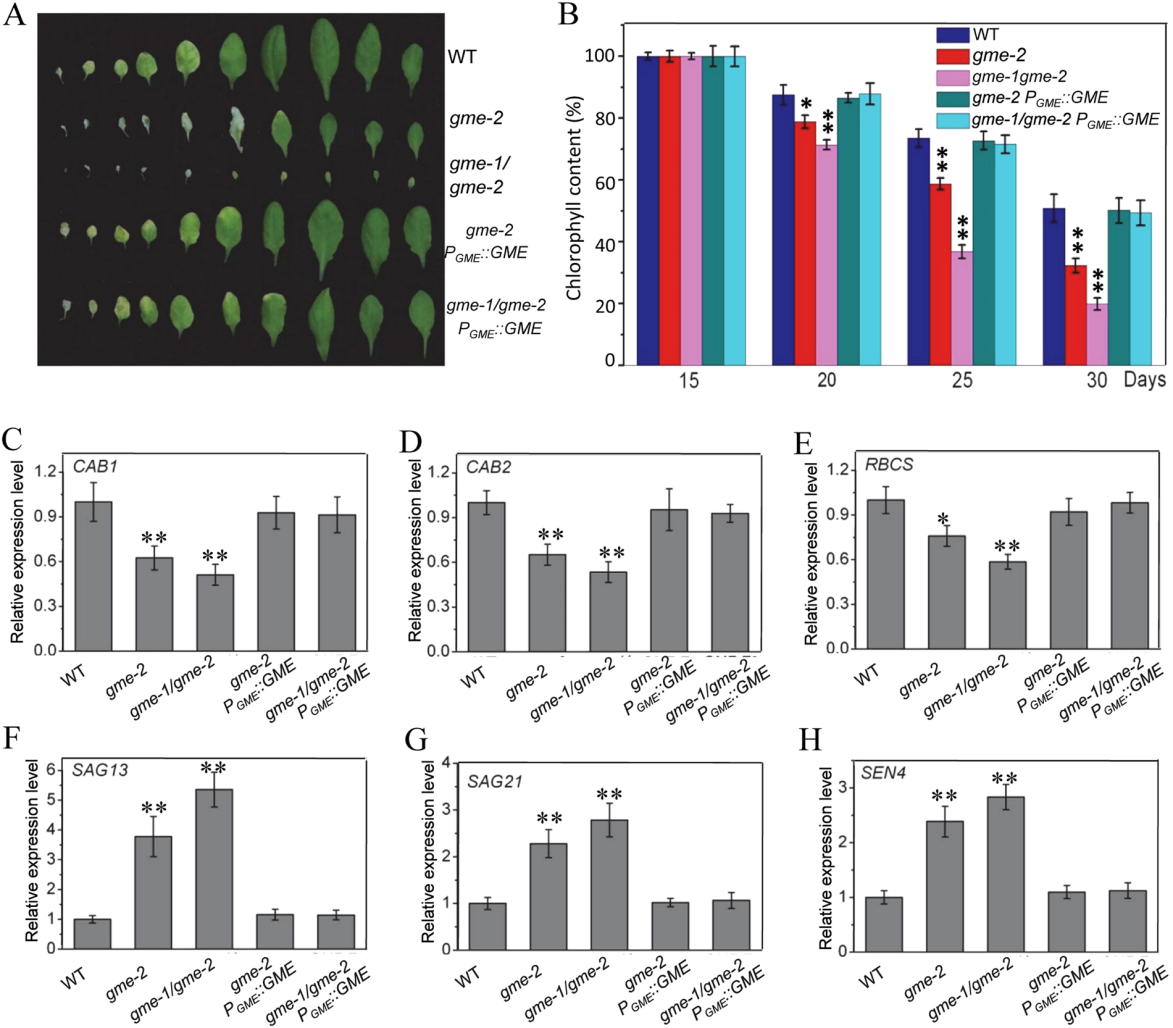
The *gme* mutants exhibited early leaf senescence. (**A**) The leaves of 6-week-old WT, *gme-2*, *gme-1*/*gme-2*, *gme-2 P*_*GME*_::*GME* and *gme-1*/*gme-2 P*_*GME*_::*GME* plants. (**B**) Relative chlorophyll content in the fifth leaf of 15-, 20-, 25- or 30-day-old WT, *gme-2*, *gme-1*/*gme-2*, *gme-2 P*_*GME*_::*GME* and *gme-1*/*gme-2 P*_*GME*_::*GME* plants. Error bars represent the SE (n = 3). *t*-test: *P < 0.05, **P < 0.01. (**C–H**) Real-time PCR analysis of the expression levels of *CAB1* (**C**), *CAB2* (**D**), *RBCS* (**E**), *SAG13* (**F**), *SAG21* (**G**) and *SEN4* (**H**). *ACTIN2* was used as an internal control. Error bars represent the SE (n = 3). Asterisks represent Student’s *t*-test significance compared with wild type (*P < 0.05, **P < 0.01).

Further observation showed that *gme-2* exhibited early senescence compared with wild type, while *gme-1*/*gme-2* displayed a much more severe early senescence phenotype (Fig. 7A). As a decreased chlorophyll content is a typical physiological marker for senescence in plants, we measured the chlorophyll contents of our *gme* mutants. As shown in Fig. 7B, the chlorophyll contents of 15-day-old *gme-2* and *gme-1*/*gme-2* plants were similar to that in wild type, but the levels decreased more quickly than in wild type at later stages of growth (e.g., days 20, 25 and 30).

We next examined the expression of senescence-reduced marker genes, including *CHLOROPHYLL A*/*B-BINDING PROTEIN 1* (*CAB1*), *CAB2* and *RUBISCO SMALL SUBUNIT* (*RBCS*), and three senescence-induced genes, *SENESCENCE-ASSOCIATED GENE 13* (*SAG13*), *SAG21* and *SENESCENCE 4* (*SEN4*)^29–33^, in WT and *gme* mutant plants. Consistent with the observed physiological phenotype, the expression of *CAB1*, *CAB2* and *RBCS* was obviously down-regulated in *gme-2* and *gme-1*/*gme-2* (Fig. 7C–E), whereas the expression of *SAG13*, *SAG21* and *SEN4* was up-regulated in *gme-2* and *gme-1*/*gme-2* (Fig. 7F–H).

Further analysis showed that *GME* could rescue the chlorophyll level and expression of *CAB1*, *CAB2*, *RBCS*, *SAG13*, *SAG21* and *SEN4* in *gme-2* and *gme-1*/*gme-2* (Fig. 7). Taken together (Fig. 7), these results suggest that *GME* affects leaf senescence.

## Discussion

GME converts GDP-D-mannose to GDP-L-Gal and GDP-L-gulose, which are intermediates of L-ascorbate biosynthesis^13–15^. GDP-L-Gal is also a precursor of the cell wall polysaccharide RGII^16, 17^. Previous studies showed that the knock-down of both tomato *GME*s (*SIGME1* and *SIGME2*) by RNAi increased the level of mannose, decreased the contents of the precursors Gal and L-ascorbate, reduced the amount of RGI galactan side chains and down-regulated the cross-linking of RGII and methyl esterification of pectins in stems, resulting in retarded plant growth, leaf bleaching, fragility and reduced fruit size^19, 28^. *SIGME1* and *SIGME2* control reproductive development and vegetative growth separately^34^. In this study, through analysis of various T-DNA insertional mutants of Arabidopsis G*ME*/*gme-1*, *GME*/*gme-2*, *gme-2*, *gme-1*/*gme-2*, and the transgenic complementation lines *gme-1 P*_*GME*_::*GME*, *gme-2 P*_*GME*_::*GME* and *gme-1*/*gme-2 P*_*GME*_::*GME*, we show that *GME* controls male gametophyte transmission, plant growth and senescence in *Arabidopsis*.

Although both tomato *SIGME1* and *Arabidopsis GME* control male gametophyte development, they control different stages of male reproductive development. Firstly, *SIGME1-RNAi* plants exhibited reduced pollen fertility^34^, while pollen grains containing *gme-1* from the heterozygote *GME*/*gme-1* failed to germinate and transmit (Figs 2 and 3, and Table 1), demonstrating that *Arabidopsis GME* controls haploid gametophyte development. Secondly, pollen grains carrying the *gme-1* mutation developed to the tricellular stage but were unable to germinate; in comparison, pollen grains carrying the *gme-2* mutation could germinate, but they produced short pollen tubes (Figs 2 and 3). On the other hand, *SIGME1*-*RNAi* resulted in a reduced density of pollen grains, which usually arrested at the tetrad stage but displayed germination rates above 60%^34^.

Interestingly, the defects in pollen germination and pollen tube elongation were not rescued by application of L-ascorbate or GDP-L-Gal in the *gme* mutants (Fig. 4 and Supplemental Fig. 4), even though boric acid was able to rescue the *in muro* cross-linking capacity of cell wall polysaccharides and restore the growth defects of the *Arabidopsis gme-1*/*gme-2* mutant (Fig. 6). Consistently, the absence of ascorbate (in the *vtc2 vtc5* mutant) also had no effect on male gametophyte transmission (Supplemental Table 1). These findings suggest that *GME* regulates pollen germination and pollen tube elongation independent of both ascorbate biosynthesis and the *in muro* cross-linking of cell wall polysaccharides. The formation and modification of cell wall pectins, including RGI and RGII, affect pollen tube elongation^35–37^. It would be interesting to analyse the cell wall components of pollen grains from *gme* mutant plants in order to identify those components that are essential for GME-regulated pollen germination and pollen tube elongation.

*Arabidopsis GME* was expressed in diverse tissues, including roots, stems, leaves, flowers, pollen grains and pollen tubes (Fig. 1). Consistently, the *gme* mutants exhibited various growth defects, including reduced rosette leaf size, dwarfism, thinner stems, short siliques, reduced numbers of seeds in siliques and defects in pollen germination and pollen tube elongation (Figs 3, 5 and 6). The defects in vegetative growth of the *gme* mutants could be suppressed by the application of boric acid, but not L-ascorbate (Fig. 6), demonstrating that GDP-L-Gal is essential for GME-regulated plant growth, consistent with previous studies in tomato^19, 28^. However, the retarded growth of the *vtc2 vtc5* double mutant, another ascorbate biosynthesis mutant, could be rescued by the application of L-ascorbate, but not boric acid (Supplemental Fig. 5), suggesting that L-ascorbate is also essential for plant growth. This discrepancy between *gme* and *vtc2 vtc5* plants requires further study.

## Methods

### Plant materials and growth conditions

The *Arabidopsis thaliana* T-DNA insertion lines CS827235 (*gme-1*), SALK_008960 (*gme-2*), CS876707 (*vtc2-2*), SALK_135468 (*vtc5-2*)^2^ and *qrt1* ^27^ were obtained from the Arabidopsis Biological Resource Center (ABRC, Columbus, OH). The mutants *GME*/*gme-1 qrt*/*qrt* and *vtc2-2 vtc5–2* were generated by crossing. Pollen grains from *gme-2* were used to pollinate GME/gme-1 to generate the *gme-1*/*gme-2* mutant, and one-half of the progeny of *gme-1*/*gme-2* were *gme-1*/*gme-2*. The primers used for the verification of *gme-1*, *gme-2*, *vtc2-2* and *vtc5-2* are listed in Supplemental Table 2. The primer pairs *gme-1* (CS827235)-LP1/SAILB3, *gme-2* (SALK_008960)-RP2/SalkLBb1.3, *vtc2-2* (CS876707)-RP/SAILB3 and *vtc5-2* (SALK_135468)-RP/SalkLBb1.3 were used to verify the T-DNA insertions in *gme-1*, *gme-2*, *vtc2-2* and *vtc5-2*, respectively. *Arabidopsis* seeds were disinfected with bleach, plated on Murashige and Skoog (MS) medium and transferred to a greenhouse under a 16-h-light (22–24 °C)/8-h-dark (17–19 °C) photoperiod after being chilled for 3 days at 4 °C. For the boric acid, AsA and L-Gal supplementation assays, *Arabidopsis* seeds were disinfected and plated on boric acid-free MS medium supplemented without or with ASA, L-Gal and boric acid, respectively, for about 3 weeks, then the phenotypes of the seedlings were recorded.

### Pollen analysis

Pollen grains at floral stage 13 were harvested for morphological analysis by environmental scanning electron microscopy (FEI Quanta 200; FEI Co., Hillsboro, OR), stained with a DAPI solution (0.1 M sodium phosphate, pH 7, 0.4 µg/mL of DAPI, 1 mM EDTA and 0.1% Triton X-100) for the observation of pollen nuclei by fluorescence microscopy, Alexander staining solution^38^ or 0.5 µg/µL of fluorescein diacetate and 1 µg/µL of propidium iodide for pollen viability testing and with 0.02% neutral red for vacuole analysis.

Aniline blue staining of germinated pollen grains in pistils was performed as described previously^39^. Pollinated pistils were collected 16 h after pollination, fixed in a solution of ethanol:acetic acid (3:1) for 2 h, washed with distilled water three times, further softened with 8 M NaOH overnight and then washed with distilled water three times. The softened pistils were incubated with an aniline blue solution (0.1% aniline blue in 0.1 M K_2_HPO_4_-KOH buffer, pH 11) for about 3 h in the dark, and then observed with a Zeiss fluorescence microscope (LSM710; Carl Zeiss AG, Oberkochen, Germany).

### *In vitro* pollen germination assay

*In vitro* pollen germination assays were performed as described previously^40^ with modifications. Pollen grains were collected from flowers that had been dehydrated at room temperature for about 1 h, spread on the surface of agar medium (0.01% boric acid, 5 mM CaCl_2_, 5 mM KCl, 1 mM MgSO_4_, 10% sucrose and 1.5% low-melting agarose, pH 7.5), germinated at 22–24 °C for 12 h and then observed under a light microscope with a CCD imaging system. At least 500 pollen grains of each genotype were analysed for pollen germination rate and pollen tube length. The pollen germination medium was added with the indicated concentrations of boric acid, ASA and GDP-L-Gal sodium salt to test their effects on *gme* mutant pollen germination.

### GUS staining

The −2990 bp promoter region of *GME* was amplified from *Arabidopsis* genomic DNA and inserted into pBI121 using *Hin*dIII and *Xba*I to generate *P*_*GME*_ *::GUS*. The primers used to generate the construct are listed in Supplemental Table 2. The construct was transformed into *Arabidopsis* by the *Agrobacterium*-mediated floral dip method. Histochemical staining for GUS activity in the *P*_*GME*_ *::GUS* transgenic plants was performed as described previously^41^.

### Generation of *GME* transgenic plants

The *P*_*GME*_ *::GME* construct was generated by replacing the *GUS* gene in *P*_*GME*_ *::GUS* with the coding sequence of *GME*. The primers used to generate the construct are listed in Supplemental Table 2. The *P*_*GME*_ *::GME* construct was introduced into *GME*/*gme-1* and *GME*/*gme-2* heterozygous plants by the *Agrobacterium*-mediated floral dip method to generate *P*_*GME*_ *::GME* homozygous transgenic plants in *gme-1* and *gme-2* backgrounds. Next, *gme-1 P*_*GME*_ *::GME* was crossed with *gme-2 P*_*GME*_ *::GME* to generate *gme-1*/*gme-2 P*_*GME*_ *::GME*.

### Subcellular localisation of GME

The coding sequence of *GME* was cloned into pEGAD for fusion with *GFP*. *Agrobacterium* cells containing pEGAD or pEGAD-GME were incubated, harvested, resuspended in infiltration buffer (0.2 mM acetosyringone, 10 mM MES and 10 mM MgCl_2_), infiltrated into *Nicotiana benthamiana* leaves with a needleless syringe^42^ and incubated at 24 °C for about 50 h before observation for GFP fluorescence. The coding sequence of *GME* was cloned into pEZS to fuse it with *GFP*. *Arabidopsis* protoplasts were transformed with pEZS or pEZS-GME as described previously^43^ and observed for GFP fluorescence with a Zeiss microscope (LSM710; Carl Zeiss AG). The primers used to generate the constructs are listed in Supplemental Table 2.

### Ascorbate content measurement

Leaves of 5-week-old *Arabidopsis* plants were homogenised in 6% TCA (approximately 0.2 g FW mL^−1^) and centrifuged at 12,000 x *g* for 5 min. The ascorbate and dehydroascorbate contents were determined by iron (III) reduction^44^. Total ascorbate represents the sum of the ascorbate and dehydroascorbate contents.

### Chlorophyll content measurement

For chlorophyll content measurement, the fifth leaves of WT, *gme-2*, *gme-1*/*gme-2*, *gme-2 P*_*GME*_ *::GME* and *gme-1*/*gme-2 P*_*GME*_ *::GME* plants at different growth stages (15, 20, 25 and 30 days) were harvested and measured as described previously^45^.

### qRT-PCR and RT-PCR analyses

For the analysis of *GME* expression in different plant tissues, roots, stems, rosette leaves, stem leaves and flowers from ~5-week-old *Arabidopsis* plants were harvested for RNA extraction, reverse transcription and subsequent qRT-PCR and RT-PCR analyses. For the qRT-PCR analysis of senescence-associated genes, leaves from 6-week-old *Arabidopsis* plants were harvested and used for real-time PCR. For the qRT-PCR analysis of *GME* in WT and *gme* mutant plants, 4-week-old plants and pollen grains from plants at floral stage 13 were collected, respectively. qRT-PCR analyses were performed with an ABI 7500 real-time PCR system as described previously^46^. The primers used for qRT-PCR and RT-PCR are listed in Supplemental Table 2. *ACTIN2* was used as a normalisation or internal control.

### Accession numbers

The *Arabidopsis* Genome Initiative numbers for the genes mentioned in this article are as follows: *GME* (AT5G28840), *ACTIN2* (AT3G18780), *QRT1* (AT5G55590), *VTC2* (AT4G26850), *VTC5* (AT5G55120), *CAB1* (AT1G29930), *CAB2* (AT1G29920), *RBCS* (At1g67090), *SAG13* (AT2G29350), *SAG21* (AT4G02380) and *SEN4* (AT4G30270).

### Data availability

All data generated or analysed during this study are included in this published article and the Supplementary Information files.

## Electronic supplementary material

Supplemental Information
