# Supplementary material for: GDP-D-mannose epimerase regulates male gametophyte development, plant growth and leaf senescence in Arabidopsis
Source: Sci Rep. 2017 Sep 4;7:10309. doi: 10.1038/s41598-017-10765-5 (PMC5583398; doi:10.1038/s41598-017-10765-5)
Supplement: Supplementary file 1 — Supplemental Information [file 41598_2017_10765_MOESM1_ESM.pdf]

Supplementary Information

GDP-D-mannose epimerase regulates male gametophyte development, plant growth and leaf senescence in *Arabidopsis*

Tiancong Qi[1], Zhipeng Liu[2], Meng Fan[1], Yan Chen[3], Haixia Tian[3], Dewei Wu[1], Hua Gao[1], Chunmei Ren[3]*, Susheng Song[4]*, Daoxin Xie[1,]*

1 Tsinghua-Peking Center for Life Sciences, MOE Key Laboratory of Bioinformatics, School of Life Sciences, Tsinghua University, Beijing 100084, China.

2 State Key Laboratory of Grassland Agro-ecosystems, College of Pastoral Agriculture Science and Technology, Lanzhou University, Lanzhou 730020, China.

3 College of Bioscience and Biotechnology, Crop Gene Engineering Key Laboratory of Hunan Province, Hunan Agricultural University, Changsha, Hunan 410128, China.

4 Beijing Key Laboratory of Plant Gene Resources and Biotechnology for Carbon Reduction and Environmental Improvement, College of Life Sciences, Capital Normal University, Beijing 100048, China.

*Correspondence: daoxinlab@tsinghua.edu.cn, songsslab@163.com, or rencm66@163.com

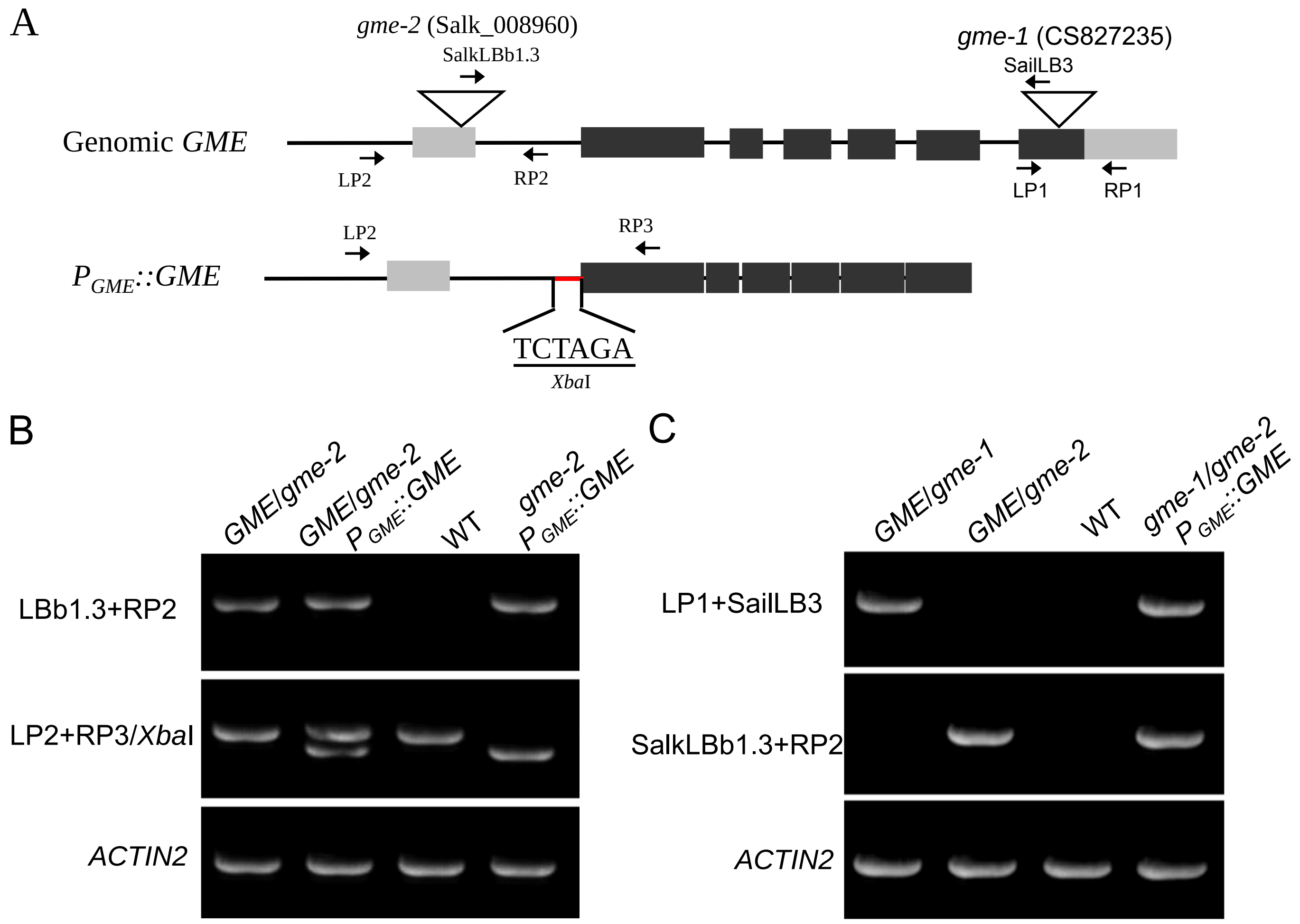

Supplemental Figure 1. Genotyping of *gme* mutants and complementary transgenic lines.

(A) Schematic diagrams showing the T-DNA insertion site in *gme-2*, and the P_{GME}::*GME* vector. The grey rectangle, black rectangle and triangle represent the UTR, exon, and T-DNA insertion site, respectively. The red line indicates the inserted artificial sequence in the P_{GME}::*GME* vector. The primers indicated by arrows were used to identify the mutant background in (B) and (C).

(B) PCR to identify the genetic background of the *GME*/*gme-2*, *GME*/*gme-2* P_{GME}::*GME*, WT and *gme-2*/*gme-2* P_{GME}::*GME* plants. The top panel shows the PCR products for the *gme-2* T-DNA insertion using primers RP2 and LBb1.3. The middle panel shows the results of the digestion of the PCR products amplified by primers LP2 and RP3 with *Xba*I. The bottom panel shows the PCR products for *ACTIN2* as a control.

(C) PCR to identify the genetic background of the *GME*/*gme-1*, *GME*/*gme-2*, WT and *gme-1*/*gme-2* P_{GME}::*GME* plants. The top and middle panels, respectively, show the PCR products for the *gme-1* T-DNA insertion (LP1 and LB3) and *gme-2* T-DNA insertion (LP2 and LBb1.3). The bottom panel shows the PCR products for *ACTIN2* as a control.

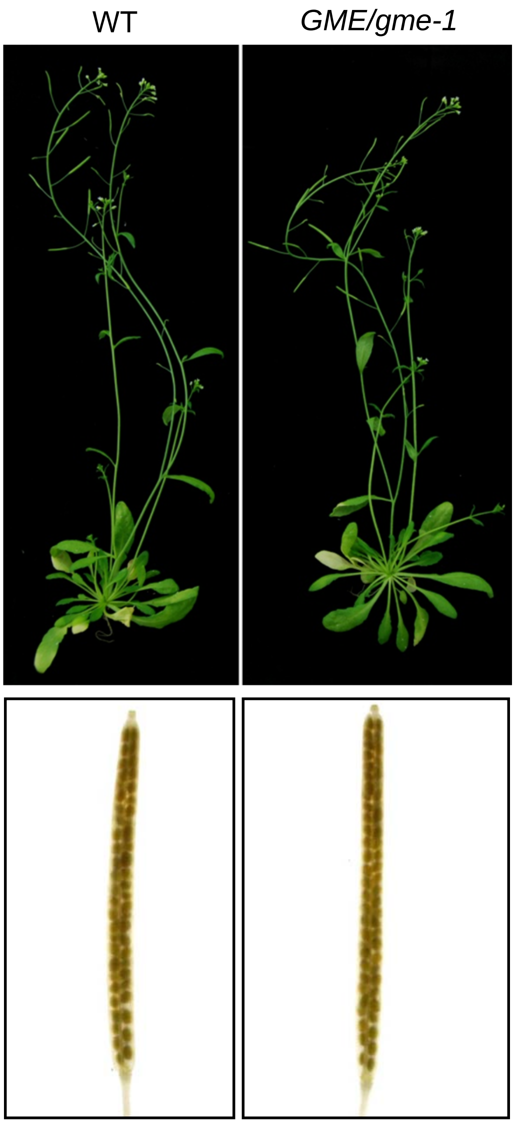

Supplemental Figure 2. Phenotype of a *GME/gme-1* heterozygous plant.

Six-week-old WT and *GME/gme-1* plants (top). The siliques were bleached with ethanol and imaged (bottom). As shown, the *GME/gme-1* plant grew normally and exhibited similar fertility to wild type.

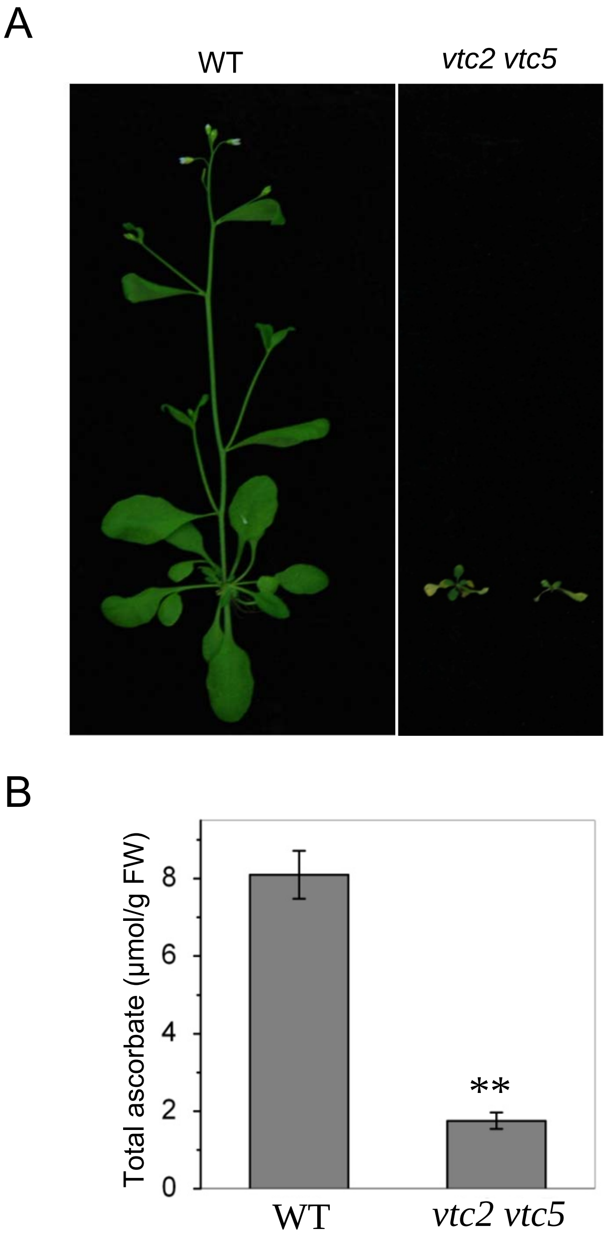

Supplemental Figure 3. Plant phenotype and total ascorbate content in the *vtc2 vtc5* double mutant.

(A) Morphology of 5-week-old WT and *vtc2 vtc5* double mutant plants.

(B) Total ascorbate contents in the leaves of 4-week-old Col-0 WT and *vtc2 vtc5* plants. Error bars represent the SE (n=3). Asterisks represent Student's *t*-test significance compared with wild type (*P<0.05, **P<0.01).

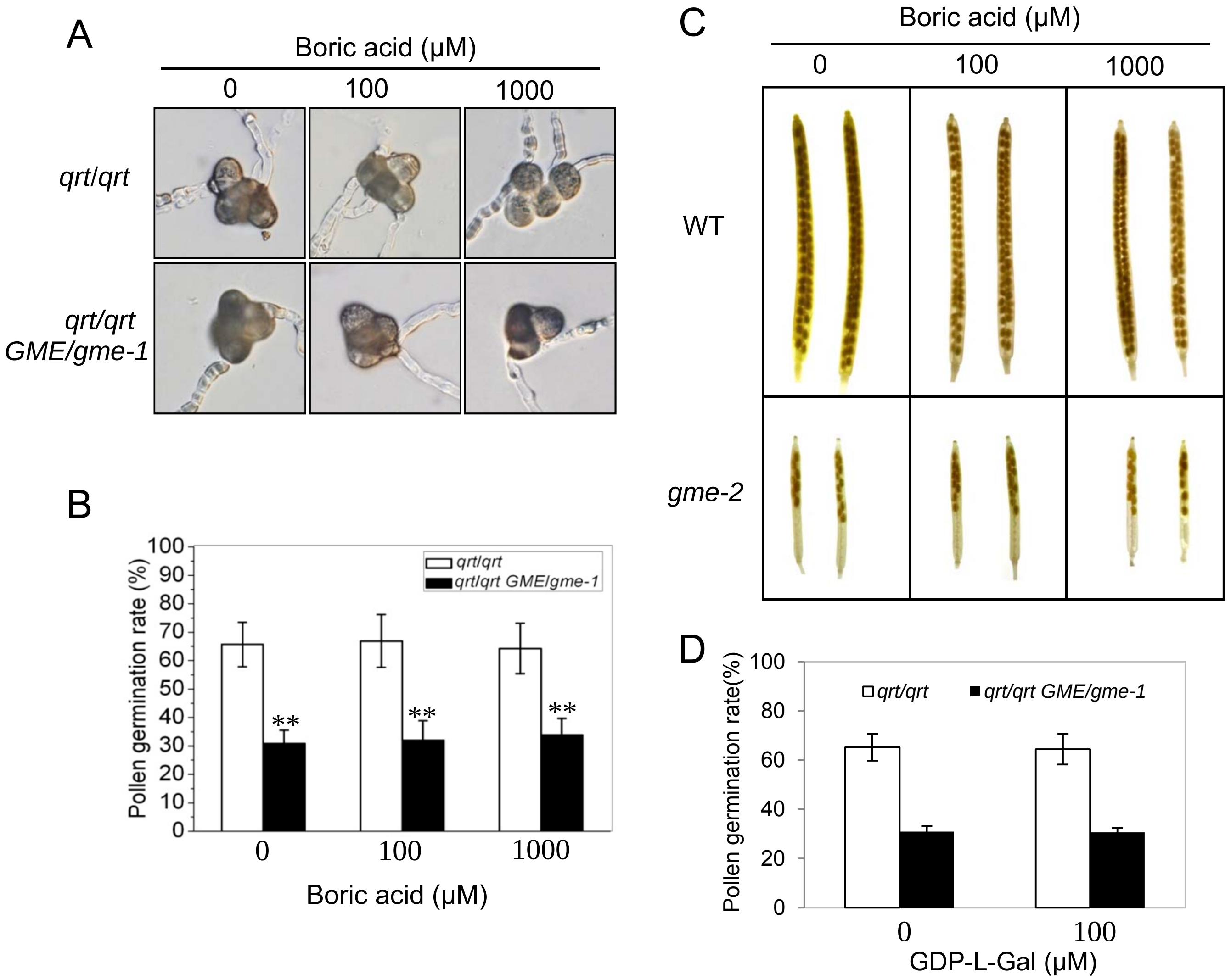

Supplemental Figure 4. Treatment with boric acid or GDP-L-Gal could not rescue the fertility of the *gme* mutant.

(A and B) *In vitro* pollen germination (A) and the pollen germination rates (B) of *qrt/qrt* and *qrt/qrt GME/gme-1* on pollen germination medium containing the indicated concentrations of boric acid. Error bars represent the SE (n=3).

(C) Inflorescences of 5-week-old WT and *gme-2* plants were treated with 0, 100 or 1000 μM boric acid for 10 days. The siliques were derived from flower buds of WT and *gme-2* plants treated with 0, 10 or 100 μM boric acid. Asterisks represent Student's *t*-test significance compared with *qrt/qrt* (**P<0.01).

(E) The pollen germination rates of *qrt/qrt* and *qrt/qrt GME/gme-1* on pollen germination medium containing the indicated concentration of GDP-L-gal sodium salt. Error bars represent the SE (n=3).

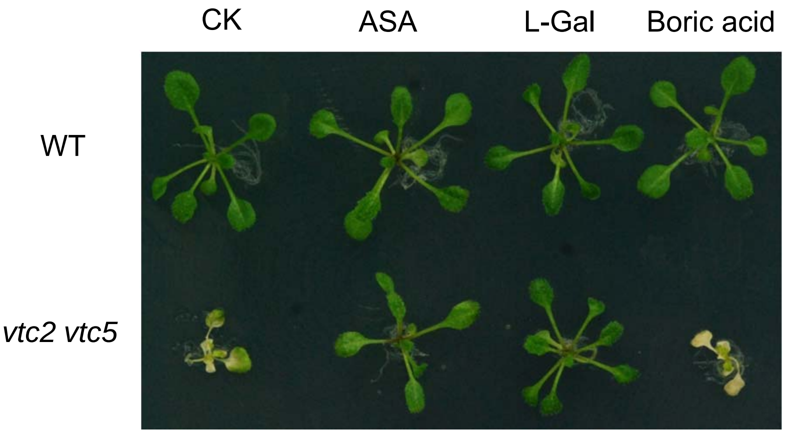

Supplemental Figure 5. The growth of *vtc2 vtc5* could be rescued by treatment with ASA or L-Gal.

Three-week-old seedlings of Col-0 WT and *vtc2 vtc5* double mutant plants grown on boric acid-free MS medium treated with mock, 100 µM ASA, 100 µM L-Gal or 100 µM boric acid.

Supplemental Table 1. Genetic transmission analysis of *vtc2* and *vtc5* alleles

Genotype	T-DNA$^+$	T-DNA$^-$	TE(%)
♀ WT × ♂ *VTC2/vtc2 vtc5/vtc5*	134	141	48.7
♀ *VTC2/vtc2 vtc5/vtc5* × ♂WT	107	101	51.4
♀ WT × ♂ *vtc2/vtc2 VTC5/vtc5*	103	108	48.8
♀ *vtc2/vtc2 VTC5/vtc5* × ♂WT	112	108	50.9

Progeny containing a T-DNA insertion (*vtc2* for the top two panels and *vtc5* for the bottom two panels) were identified by PCR. Transmission efficiency (TE)=Number of progeny with a T-DNA insertion/Number of progeny×100%. WT, Col-0 wild type.

Supplemental Table 2. Primers used for identification of *gme* T-DNA mutants and generation of constructs.

gme-1(CS827235)-LP1	GACAAAATCAGATTTCCGTG
gme-1(CS827235)-RP1	GCTGAACTTAAAAGGCCATATTTG
gme-1(CS827235)-SAILB3	TAGCATCTGAATTTCATAACCAATCTCGATACAC
gme-2(SALK_008960)-LP2	TTCCTCTTCCTCAAATCTCTG
gme-2(SALK_008960)-RP2	AGAGTTTTGACAGAGGTAAG
gme-2(SALK_008960)-SalkLBb1.3	ATTTTGCCGATTTCGGAAC
GME-LP2	TTCCTCTTCCTCAAATCTCTG
GME-RP3	AATCACAGAGTGATTACTCTG
GME pro-HindIII-Forward	CCCAAGCTTGATCAAGATGACTAATTTAAC
GME pro-XbaI-Reverse	CTAGTCTAGATCTACATTAACAAAACAAATCTG
GME-XbaI-Forward	TGCTCTAGAATGGGAACTACCAATGGAACAG
GME-SacI-Reverse	ATCGAGCTCTCACTCTTTTCCATCAGCCGC
35S::GME-GFP-EcoRI-Forward	CCGGAATTCATGGGAACTACCAATGGAACAGACTATG
35S::GME-GFP-BamHI-Reverse	CGCGGATCCTCACTCTTTTCCATCAGCCGC
35S::GME-GFP-XhoI-Forward	ACCGCTCGAGATGGGAACTACCAATGGAAC
35S::GME-GFP- EcoRI-Reverse	GGGGAATTCGCTCTTTTCCATCAGCCGCGC
GME-Realtime-Forward	GGAGCTGGAGGTTTCATTGC
GME-Realtime-Reverse	CGTAATGACCTTCGTGCTTCAA
GME-RT-Forward	ATGCTTCGAGTGCTTGTATC
GME-RT-Reverse	GAAAGCAGCTGGAGCCTTCTC
vtc2-2(CS876707)-LP	CGGAGAGGTAAATCATAGAC
vtc2-2(CS876707)-RP	CCATACTCTATCGGACTAAC
vtc2-2(CS876707)-SAILB3	TAGCATCTGAATTTCATAACCAATCTCGATACAC
vtc5-2(SALK_135468)-LP	GCTGAAGCCGATAATCCGTA
vtc5-2(SALK_135468)-RP	CCTCCATGCCTTCTCTTCTG
vtc5-2(SALK_135468)-SalkLBb1.3	ATTTTGCCGATTTCGGAAC
CAB1-Realtime-Forward	GCAAGGAACCGTGAACTAGAA

CAB1-Realtime-Reverse	TCCGAACTTGACTCCGTTTC
CAB2-Realtime-Forward	TCAATCTTTTGAATTCGAGTGAGA
CAB2-Realtime-Reverse	TCCACCACAAACACAAACCTAC
RBCS-Realtime-Forward	CGCTCCTTTCAACGGACTTA
RBCS-Realtime-Reverse	AGTAATGTCGTTGTTAGCCTTGC
SAG13-Realtime-Forward	AGGAAAACTCAACATCCTCGTC
SAG13-Realtime-Reverse	GCTGACTCGAGATTTGTAGCC
SAG21-Realtime-Forward	CATTCCTCGGATATATCTCTCCTT
SAG21-Realtime-Reverse	TTCTTGAAAAGAGAGAAAACAGACTTT
SEN4-Realtime-Forward	AAGGTGACAAAGAGCAACAATTC
SEN4-Realtime-Reverse	CTCTCTAATGGGTGTGTCATCG
ACTIN2-RT-Forward	GCACCCTGTTCTTCTTACCG
ACTIN2-RT-Reverse	AACCCTCGTAGATTGGCACA
ACTIN2-Realtime-Forward	CCGCTCTTTCTTTCCAAGC
ACTIN2-Realtime-Reverse	CCGGTACCATTGTCACACAC
